# Association of Healthy Eating Index-2015 and Dietary Approaches to Stop Hypertension Patterns with Insulin Resistance in Schoolchildren

**DOI:** 10.3390/nu14204232

**Published:** 2022-10-11

**Authors:** María Dolores Salas-González, Aranzazu Aparicio, Viviana Loria-Kohen, Rosa M. Ortega, Ana M. López-Sobaler

**Affiliations:** 1 VALORNUT Research Group, Department of Nutrition and Food Science, Faculty of Pharmacy, Complutense University of Madrid, 28040 Madrid, Spain; 2 VALORNUT Research Group, Department of Nutrition and Food Science, Faculty of Pharmacy, Complutense University of Madrid, IdISSC, 28040 Madrid, Spain

**Keywords:** children, insulin resistance, HEI, DASH

## Abstract

Background: Diet quality patterns are associated with a lower incidence of insulin resistance (IR) in adults. The aim of this study was to investigate the association between two diet quality indices and IR in schoolchildren and to identify the best diet quality index associated with a lower risk of IR. Methods: A total of 854 schoolchildren (8–13 years) were included in a cross-sectional study, who completed a three-day dietary record to assess their diet. Fasting plasma glucose and insulin were also measured, and anthropometric data were collected. Healthy Eating Index-2015 (HEI-2015), Dietary Approaches to Stop Hypertension (DASH), and adjusted DASH (aDASH) were calculated as diet quality indices. The homeostasis model assessment of insulin resistance (HOMA-IR) was used, and IR was defined as HOMA-IR > 3.16. Results: The prevalence of IR was 5.5%, and it was higher in girls. The mean HEI-2015 and DASH scores were 59.3 and 23.4, respectively, and boys scored lower in both indices. In girls, having a HEI-2015 score above the 33rd percentile was associated with a lower risk of IR (odds ratio [95% CI]: 0.43 [0.19–0.96], *p* = 0.020). Conclusion: Greater adherence to a healthy dietary pattern, as assessed by a higher HEI-2015 score, was associated with a lower risk of IR in schoolchildren, especially in girls.

## 1. Introduction

With the growing epidemic of obesity, incidence of insulin resistance (IR) in childhood and adolescence has increased worldwide. Moreover, IR is accompanied by other components of metabolic syndrome and continues into adulthood [1].

IR is a common manifestation of obesity. Initially, pancreatic beta cells are able to compensate for IR by increasing insulin secretion in the pathogenesis of glucose intolerance. Compensatory hyperinsulinemia induces an increase in appetite, and consequently, weight gain. After pancreatic beta cell function decreases, insufficient insulin secretion occurs, leading to a transition from IR to impaired glucose tolerance, followed by type 2 diabetes [2,3].

Diet is one of the main modifiable factors that can help control and prevent obesity-associated disorders such as IR. Several scores and indices are available to assess the diet quality or the adherence to a healthy dietary pattern. They are a multidimensional representation of diet and do not focus on specific nutrients or foods. Adherence to healthier dietary patterns has been associated with lower risk of several diseases, such as cardiovascular disease, type 2 diabetes, metabolic syndrome, and some of its characteristics, such as IR, in both children and adults. For example, a healthier diet was associated with lower mortality [4], better cardiovascular health [5], and lower risk of metabolic syndrome [6], type 2 diabetes [7], hypertension [8] or IR in adults [9,10]. Moreover, there are some studies in children that have observed that a higher adherence to a healthy dietary pattern was associated with better cardiovascular health [11] and lower risk of metabolic syndrome [12,13], hypertension [14], or IR [15,16], although in children, the evidence is still very scarce.

A number of indices have been proposed to assess adherence to a Mediterranean dietary pattern. However, some have been designed for application in the adult population, such as the Mediterranean Diet Adherence Screener (MEDAS) of the PREDIMED study [17] and are not applicable in children. Others, such as the Mediterranean Diet Score (MDS) developed by Trichopoulou et al. [18], have been subsequently modified to include fish consumption [19] and/or has been adapted to children [20,21]. However, the MDS has a small range (0–9 points), and more than half of the population scored between 3 and 5, which suggests that MDS may not be able to distinguish between individuals with different patterns of dietary intake [22]. On the other hand, in Spain, there is no consensus on dietary recommendations or guidelines for Spain, so there is no validated diet quality index for the Spanish population.

The Dietary Approaches to Stop Hypertension (DASH) [23] and the Healthy Eating Index (HEI), updated in 2015 (HEI-2015) [24,25] are some of the most widely used diet quality indices to assess adherence to dietary guidelines in Americans. However, many studies worldwide have used it [14,26,27]. Spain is one of the countries in which the DASH and HEI have been used in both adult and child populations [28,29,30]. The use of diet quality indices worldwide is feasible due to the similarity of recommendations and allows comparison of the results of the relationship between insulin resistance and diet.

According to a systematic review [31] on the epidemiology of IR in children, most studies focus on adolescence, and few studies (four out of 18) focused on children younger than 10 years. This could be due to the fact that the insulin resistance peaks at mid-puberty [32] and most studies have focused on this age group. Nevertheless, Chiarelli et al. [33] point out that studying the IR in prepubertal children is especially important, as IR and related complications could be exacerbated by the influence of puberty, due to the physiological decrease in insulin sensitivity associated with normal pubertal development.

However, there are few studies on diet quality indices and IR in children, and, therefore, the possible relationship between them needs to be investigated. This study aimed to investigate the association of different diet quality indices with IR in schoolchildren and to identify the diet quality index associated with a lower risk of IR. This study could help identify appropriate measures for use in epidemiological studies and improve the success of nutritional measures and interventions by targeting significant dietary components in at-risk groups.

## 2. Materials and Methods

### 2.1. Study Design and Sample

The cross-sectional observational study included a convenience sample of schoolchildren aged 8–13 years from five Spanish provinces (A Coruña, Barcelona, Madrid, Seville, and Valencia). In each province, schools were randomly selected from a list of primary schools with at least two classrooms per grade. The principals of 55 schools were contacted by telephone to explain the characteristics and importance of the study, and to request the school’s permission to participate in the survey. Thirteen principals did not want to collaborate. The principals of the remaining 42 schools showed interest in the study and forwarded the information to the school council for approval. Twenty-two schools gave their permission to conduct the survey on their premises and to organize a meeting with the parents of the children in the target age range. At this meeting, the study was explained in detail, questions raised by the parents were answered, and signed parental authorization for the children to take part was requested.

**Figure 1:**
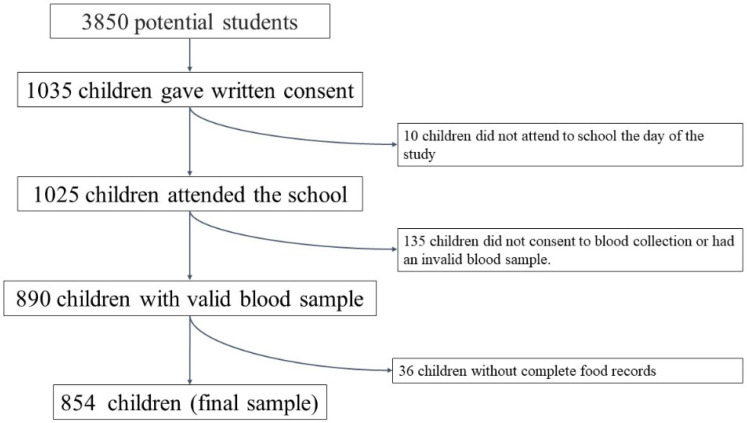
Flow chart of the selection process.

The potential initial sample size was approximately 3850 participants. These children were studied previously in another context [34]. It was calculated taking into account the number of schools that agreed to participate (*n* = 22), the number of students per classroom (*n* = 25) and the number of classrooms per grade (between 2 and 3). Subsequently, 1035 schoolchildren obtained written consent from their parents or guardians to participate in the study, so the approximate acceptance rate was 27%. Finally, 854 students completed all the dietary questionnaires and the anthropometric and biochemical study. Figure 1 shows a flow chart of the sample selection process.

The inclusion criteria were as follows: boys and girls aged 8–13 years who were in 4th, 5th, and 6th grades of primary education; written informed consent signed by the child’s parents and/or guardians; and acceptance of all the study conditions.

The following exclusion criteria were applied: the presence of any disease, such as metabolic or chronic diseases (diabetes, renal diseases, etc.) that could affect the study outcomes, inability to visit the study centre on the agreed days, and pharmacological treatment, such as corticosteroids, insulin, and levothyroxine, that could interfere with study outcomes.

The participating schoolchildren underwent a dietary, anthropometric, and biochemical study at school by trained investigators. Measurements were made between February 2005 and June 2009.

This study was conducted in accordance with the guidelines of the Declaration of Helsinki. This study was approved by the Human Research Review Committee of the Faculty of Pharmacy of the Complutense University of Madrid (PI060318 approved at 17 March 2006).

### 2.2. Anthropometric Study

All measurements were performed at schools in the morning according to the World Health Organization (WHO) standards [35].

Weight and height were measured with the participants in underwear and barefoot using a digital electronic scale (model SECA ALPHA, GMBH & Co., Igny, France) (range: 0.1–150 kg, accuracy 100 g) and a Harpenden digital stadiometer (Pfifter, Carlstadt, NJ, USA) (range: 70–205 cm, accuracy 1 mm), respectively. Body mass index (BMI) was calculated by dividing the weight (kg) by height squared (m^2^). Subsequently, the BMI z-score (z-BMI) was calculated. The cut-off points were established according to the standard deviation of the z-score established by the WHO [36] (z < −2: underweight, z: −2–1: normal weight z: 1–2: overweight; y z > 2: obesity) [37].

The tricipital skinfold was measured on the right side of the body using a Holtain skinfold caliper (constant pressure of 10 g/mm^2^ (range: 0–39 mm) and 0.1 mm accuracy, Holtain Ltd., Crymych, Wales) following the recommendations by the ISAK [38]. Fat mass (FM) was obtained using the formula by Dezemberg et al. in 1999 [39]. FM was classified according to McCarthy’s cut-off points as insufficient fat, normal fat, excessive fat, and obesity [40].

### 2.3. Physical Activity

Parents completed an adapted daily physical activity questionnaire for their children [41], which has been used in other studies [34,42,43,44].

The questionnaire asked about the time dedicated to different activities usually carried out during the day: sleeping, being in class, study time, time dedicated to different meals, time dedicated to sedentary play (with PC, video consoles or watching television); time dedicated to playing actively in the street, play time at home; time dedicated to gymnastics or sports activities at school, at recess and in extracurricular activities, time and form of travel between home and school and to other activities.

The time spent on the different activities was then grouped into 4 categories: sleep, very light activities (activities done lying, sitting or standing -painting, playing an instrument, cooking...), light activities (equivalent to walking on a flat surface at 4–5 km/h, cleaning the house, golf, table tennis…) and moderate and/or vigorous activities (physical activities requiring greater energy expenditure such as cycling, skiing, tennis, dancing, basketball, soccer, rugby, running…).

An activity coefficient was established for each subject by multiplying the time dedicated to each activity by the established coefficients [45,46]: 1 for sleeping and resting, 1.5 for very light activities, 2.5 for light activities, 5 for moderate activities, and 7 for intense activities, and then dividing by 24 h. Once the individual physical activity coefficient was obtained, it was used to establish the physical activity category for the calculation of energy expenditure (EE) according to IOM equations [47].

### 2.4. Dietetic Study

For dietary intake, a ‘food record’ was filled out for three consecutive days, including one weekend day (Sunday to Tuesday) [48]. Parents were asked to record the weights, if possible, or alternatively, to record home measurements of all foods consumed outside of school by their children. On Mondays and Tuesdays, the trained staff visited the school canteen, recorded the food on the menu, and weighed the amount of food served to each child who stayed to eat in the canteen and what each child left on the plate. DIAL software (Alce Ingeniería, Madrid, Spain) was used to process the dietary surveys [49]. The food composition tables from Ortega et al. [50] were used. Energy intake (EI) and macro- and micronutrients were calculated. Energy expenditure (EE) was calculated according to the IOM equations [47].

The possible underestimation or overestimation of EI was determined as the discrepancy between EI and EE, measured using the formula: (EE-EI) × 100/GE. This formula provides the percentage of possible underestimation (if values are positive) or overestimation of intake (when values are negative) [51].

The HEI-2015 was used to assess diet quality according to the recommendations of the U.S. Dietary Guidelines. It is composed of 13 components: 9 for adequacy and 4 for moderation. Whole grains, dairy, and fatty acid ratio items were scored 0–10; total fruits, whole fruits, vegetables, seafood and vegetable protein, green vegetables and legumes, and protein food items were scored 0–5; and moderation, refined grains, sodium, added sugars, and saturated fat items were scored 0–10 [24].

The DASH index was also calculated [23], It includes eight components that are relevant to the diet used for hypertension management. For the DASH index, scoring is based on intake quintiles created within the full data set; participants in the lowest quintile received 1 point, and individuals in quintile five received 5 points. Red and processed meat, sugar-sweetened beverages, and sodium were reverse coded.

Finally, adjusted DASH (aDASH) was calculated in the same way as DASH after adjusting the grams of the different food groups according to the energy consumed [16].

### 2.5. Biochemical Studies

Participants’ blood samples were drawn by venepuncture between 08.00 and 09.00 h after 12 h of fasting. The nurses verified the adequacy of the fasting period before sample collection. All extractions were performed at the respective centers.

Plasma glucose was determined calorimetrically using the glucose oxidase-peroxidase method [52] (Vitros GLU Slides, Rochester, NY, USA; CV = 2.8%). Fasting insulin was measured by immunochemiluminometric assay [53] (Abbott Diagnostics, Madrid, Spain; CV = 4.8%). The QUICKI index was evaluated as1/[log (basal insulin) + log (basal glucose)] [54].

The homeostasis model assessment of insulin resistance (HOMA-IR) was used to reflect the degree of IR [55,56]: HOMA-IR = [basal glucose (mmol/L) × basal insulin (μU/mL)]/22.5. In our study IR was defined as HOMA-IR > 3.16, as proposed by Keskin et al. [57] for children and adolescents, since this cut-off point showed the highest sensitivity and specificity for the diagnosis of IR assessed by an oral glucose tolerance test. This cut-off point has also been used in other studies in schoolchildren of similar age to our study [16,58,59,60,61].

### 2.6. Statistical Analysis

Descriptive data were expressed as means and standard deviations. Diets were compared according to sex and presence or absence of IR. For comparison of means, the Mann–Whitney U test was used if the distribution of the variables was not homogeneous, the Student’s *t*-test for homogeneous distributions, and the two-way ANOVA test. The χ^2^ test was used to determine the significance of the differences between proportions. Logistic regression analysis was performed to identify risk or protective factors associated with IR. Significance was set at *p* < 0.05. All calculations were performed using IBM SPSS Statistics for Windows, Version 25.0. Armonk, NY, USA: IBM Corp. Released 2017.

## 3. Results

### 3.1. Sample Characteristics

A total of 1035 schoolchildren (49.2% boys) were initially included. Ten participants did not attend school on the day of the anthropometric study and were, therefore, excluded from the analysis. Valid dietary data were only obtained from 965 schoolchildren (48.8% boys), valid blood samples from 890 participants (48.4% boys), and 854 (48.4% boys) who have both blood and dietary samples to constitute the sample of the present study.

Table 1 shows the age and anthropometric, biochemical, and lifestyle data of the total sample classified by sex. Approximately one third of the schoolchildren (27.4%) were overweight and 12.4% were obese, mostly comprising boys. In addition, 31.6% of the participants had excessive fat and 31.5% had obesity according to their fat mass, with boys having higher obesity percentage.

Regarding biochemical data, boys had higher values of glucose and QUICKI index but lower values of insulin and HOMA-IR index. A 5.3% of the sample presented IR, and this was higher in girls than in boys (7. 0% vs. 3.4%, *p* < 0.05). Table S1 shows the age and anthropometric, biochemical, and lifestyle data of the total sample classified by group of age. Table S2 shows the age and anthropometric, biochemical and lifestyle data of the total sample classified by sex and HOMA-IR. There was a relationship between HOMA-IR and age, zBMI, fat mass, and activity coefficient.

### 3.2. Diet Quality

Dietary data, diet quality data, and scores for each of the sub-items are shown in Table 2, and Table S3 shows the dietary intake of those foods and nutrients included in the different diet quality indices. Boys had higher caloric intake than girls, and the underestimation of their diet was more significant than in girls (7.65 ± 18.95% vs. −11.27 ± 23.28%). They also scored significantly lower on all three calculated quality indices (HEI-2015, DASH, and aDASH). According to the HEI-2015 sub-items, boys scored lower on servings of total fruit, whole fruit, green vegetables, and legumes. Likewise, boys scored lower on the (PUFAs+MUFAs)/SFAs ratio and sodium level than girls. Girls scored higher on red meat, sugar-sweetened beverages, sodium (according to DASH and aDASH), and vegetables and fruits (according to aDASH) than boys. Table S4 shows diet quality as a function of age and sex, with younger children having better scores in diet quality index according to allboth indices. The diets of the younger schoolchildren had a better ratio of unsaturated to saturated fatty acids and consumed more dairy products. While older children consumed more red meat, more sugary drinks, and more sodium per 1000 kcal.

### 3.3. Risk of IR

Energy expenditure was higher in girls and boys with IR versus their non-IR counterparts. There were no differences in energy or the scores of the different diet quality indices between schoolchildren with and without IR (Table 3). Girls with IR scored lower in vegetables, green vegetables, and legumes according to the HEI-2015 parameters and lower in whole grains according to the DASH score than girls without IR. Boys with IR scored lower on sugar-sweetened beverages according to the DASH and aDASH scores than did boys without IR. Table S5 shows the consumption of foods and nutrients related to the analyzed, diet quality indices subitems by sex and IR, but there were no significant differences to highlight. Table S6 shows the quality of the diet according to the HOMA-IR in the two age groups. In the younger girls group, those with IR consumed more protein and more protein products than their peers without IR, however there were no differences in the score for protein products. In the older age group, boys with IR consumed more skimmed dairy products, which make them score better on this item in the DASH and aDASH.

Table 4 presents the logistic regression analyses with tertiles of the different diet quality indices (the limits of each tertile are shown in Table S7) and the risk of IR. The results are shown in crude (Model 1) and adjusted for age, sex, z-BMI, and activity coefficient (Model 2).

For HEI-2015, in girls, having a score higher than the 33rd percentile (≥56.8 points) was associated with a lower risk of having IR (adjusted model 2: OR = odds ratio (95% confidence interval): 0.43 (0.19–0.96), *p* = 0.020), both in the crude and adjusted models. No association was found in boys. In the DASH and aDASH, no risk of IR was found according to tertiles, neither in the total sample nor by sex.

## 4. Discussion

To our knowledge, this is the first study to relate various diet quality indices to IR in children in our country.

In a sample of 890 schoolchildren, it was observed that having a higher adherence to the HEI-2015 diet-quality index was associated with a lower risk of IR in girls but not in boys. Higher adherence to the DASH diet did not appear to be related to IR in either sex.

Furthermore, the prevalence of IR in the sample was 5.5%, which is similar to that found in other age-matched populations in Greece [58] and Spain [44]. The prevalence of IR was higher in girls than in boys, and these data agree with those obtained in other studies on schoolchildren [33,62]. This could be because pubertal development occurs earlier in girls than in boys and because of the differences in hormone levels between the sexes [32,63]. Therefore, the results were also expressed by sex, and differences for both sexes were observed.

The mean diet quality assessed using the HEI-2015 was 59.28 points. These data are similar to those of other studies performed in American children [64,65]. According to our results, greater adherence to the HEI-2015 is a protective factor against IR, which is consistent with other studies conducted in adults [66]. Compared with previous versions of the HEI, Monfort-Pires et al. observed in a study of an adult population that greater adherence to the HEI decreased HOMA-IR [9]. Moreover, in a group of adult men in the US, HEI was associated with various cardiovascular parameters, including HOMA-IR [67]. However, according to the Boston Puerto Rican Health Study results, HEI was not related to any IR parameter in the adult population [68].

Several mechanisms may explain the relationship between HOMA-IR and HEI-2015 scores. One of them could be the scoring according to the consumption of plant-based foods rich in fibre. The relationship between the fibre and IR is complex. Insoluble fibres can improve postprandial satiety and reduce appetite. It also improves IR, as measured using euglycemic hyperinsulinemic clamps. Soluble fibre can trap carbohydrates and nutrients to decrease their absorption and delay gastric emptying, which would reduce the postprandial glycaemic response. In addition, it can ferment in the intestine. It may improve the quality of the gut microbiome, increasing the production of short-chain fatty acids, which would help regulate the sympathetic and parasympathetic nervous systems, in turn regulating glucose metabolism and IR [69]. The HEI-2015 also has two sub-items referring to fats and their quality. Saturated fatty acids (SFAs) are more prone to be stored in muscle than monounsaturated fatty acids and can cause increased IR in the muscle through increased intramyocellular lipid accumulation. In addition, long-chain SFAs, compared with unsaturated fats, are more readily incorporated into diacylglycerol than triacylglycerol, which may also increase inflammation and IR [70] Another reason could be the negative score for added sugars. A high intake of sugars results in an increased glycaemic load on the diet, leading to β-cell dysfunction, inflammation, and IR [71]. In the current study, the results are presented as a full index, possibly because with an adequate diet these mechanisms are jointly enhanced or complemented in some way.

In our study, the mean DASH score was 23.4, with a maximum score of 40, which is slightly lower than that of schoolchildren in Tehran (24.0 points) [16] but higher than that of children in Brazil (15.7 points) [14]. In this study, no relationship was observed between IR and the DASH diet, which differs from Rahimi et al., where a higher aDASH score was related to lower HOMA-IR values in children [16]. Likewise, in other adult studies, DASH scores were inversely related to IR [72,73,74]. Our data are in line with those of two meta-analyses performed in adults, where no clear association was observed between DASH and HOMA-IR. However, these two studies found a trend toward insulin reduction [75,76].

The absence of results with respect to DASH may be because DASH and aDASH scores are posteriori calculated indexes, that is, they were obtained using cut-off points based on our sample. Although the HEI-2015 is an a priori index, it is based on dietary recommendations. As observed in our results, many of the schoolchildren did not have a quality diet. Therefore, the diet quality indices calculated based on our data could be affected.

Due to the contradictory results in different publications, further studies on the relationship between IR and diet quality indices would be desirable. Moreover, very few studies have addressed this problem in the pediatric population. Therefore, there is a need to focus on this age group.

Finally, in our study, insulin resistance is related to age, i.e., older children have more IR, which is consistent with the insulin peak that is described in adolescence [32]. In addition, older schoolchildren score worse on diet quality indices, and consume more red meat, sugar-sweetened beverages, and sodium. This is consistent with a study conducted with the HEI-2010 where younger children had the highest overall diet quality [77].

One of the main strengths of our study was that it included a large sample size. In addition, there is little literature on IR in children in this age range, and this is one of the first studies to relate IR in children with indicators of a healthy diet. In addition, the three-day log was used to calculate the diet quality indices in our study, which is one of the most appropriate methods. One of the main limitations of the study is that being a large community-based study, Tanner stratification by a clinician was not possible and information on the pubertal status of the participating children was not included. As only the tricipital fold could be obtained in most of the participants, the method for estimating fat mass has limitations and is not the gold standard. Although the physical activity questionnaire has been used in other studies, it is not a validated questionnaire. Finally, the sample studied is a convenience sample, so the conclusions of our study should be confirmed in future research.

## 5. Conclusions

We can conclude that greater adherence to a healthy dietary pattern, as assessed by a higher HEI-2015 score, is associated with a reduced risk of IR in schoolchildren, especially in girls. Regarding adherence to the DASH diet, no relationship with IR was observed in our study. Given the above results, promoting a healthy diet based on the HEI-2015 index in this age group could help reduce IR and, therefore, prevent the development of type 2 diabetes and metabolic syndrome.

## Figures and Tables

**Table 1:** Anthropometric, physical activity and blood biochemical parameters in the schoolchildren studied according to sex.

	Total (*n* = 854)	Girls (*n* = 441)	Boys (*n* = 413)	*p*-Value
Age (years)	10.1 ± 0.9	10.2 ± 0.9	10.1 ± 1.0	0.262
8–10 years [%(*n*)]	63.35 (541)	61.7 (272)	65.1 (269)	0.295
11–13 years [%(*n*)]	36.65 (313)	38.3 (169)	34.9 (144)	
Madrid [%(*n*)]	55.6 (475)	59.0 (260)	52.1 (215)	0.237
Barcelona [%(*n*)]	7.9 (67)	7.3 (32)	8.5 (35)	
Sevilla [%(*n*)]	12.4 (106)	11.6 (51)	13.3 (55)	
A Coruña [%(*n*)]	13.4 (114)	11.3 (50)	15.5 (64)	
Valencia [%(*n*)]	10.8 (92)	10.9 (48)	10.7 (44)	
**Body composition**				
Weight (kg)	39.4 ± 9.3	39.6 ± 9.2	39.2 ± 9.5	0.432
Height (m) #	1.43 ± 0.09	1.44 ± 0.09	1.43 ± 0.08	0.008
BMI (kg/m^2^)	19.0 ± 3.1	18.9 ± 3.0	19.1 ± 3.3	0.774
Z-BMI #	0.69 ± 1.13	0.57 ± 1.04	0.81 ± 1.21	0.001
**Nutritional status by BMI**				
Underweight [%(*n*)]	0.82 (7)	0.68 (3)	0.97 (4)	<0.001
Normal weight [%(*n*)]	59.4 (507)	63.3 (279)	55.21 (228)	
Overweight [%(*n*)]	27.4 (234)	29.0 (128)	25.7 (106)	
Obesity [%(*n*)]	12.4 (106)	7.0 (31)	18.2 (75)	
Body fat (%) #	27.6 ± 5.7	29.3 ± 4.7	25.8 ± 6.1	<0.001
**Nutritional status by body fat percentage**				
Low fat [%(*n*)]	0.35 (3)	0.23 (1)	0.49 (2)	<0.001
Normal fat [%(*n*)]	36.6 (312)	40.4 (178)	32.6 (134)	
Excessive body fat [%(*n*)]	31.6 (269)	33.1 (146)	29.9 (123)	
Obesity [%(*n*)]	31.5 (268)	26.3 (116)	37.0 (152)	
**Physical activity**				
Activity coefficient	1.52 ± 0.11	1.53 ± 0.11	1.53 ± 0.11	0.006
**Biochemical data**				
Glucose (mg/dL)	84.4 ± 9.7	83.4 ± 10.0	85.5 ± 9.2	0.134
Insulin (mcU/mL)	6.3 ± 4.4	7.1 ± 4.8	5.5 ± 3.7	<0.001
QUICKI	0.38 ± 0.05	0.38 ± 0.05	0.39 ± 0.04	<0.001
HOMA-IR	1.33 ± 0.97	1.48 ± 1.08	1.17 ± 0.80	<0.001
IR [%(*n*)]	5.27 (45)	7.03 (31)	3.39 (14)	<0.001

BMI, body mass index; IR, insulin resistance. # Variable follow a normal distribution. For comparison of means, the Mann-Whitney U test was used if the distribution of the variables was not homogeneous, the Student’s *t*-test for homogeneous distributions. The χ^2^ test was used to determine the significance of the differences between proportions, *p*-values < 0.05 were considered statistically significant.

**Table 2:** Diet quality in the schoolchildren studied according to sex.

	Total (*n* = 854)	Girls (*n* = 441)	Boys (*n* = 413)	*p*-Value
Energy intake (EI) (kcal) #	2105 ± 350	2066 ± 338	2145 ± 358	<0.001
Energy expenditure (EE) (kcal)	2125 ± 377	1899 ± 265	2365 ± 325	<0.001
EI/EE (%) #	101.7 ± 22.9	110.7 ± 22.8	92.2 ± 18.7	<0.001
Proteins (% EI)	15.6 ± 2.3	15.6 ± 2.3	15.5 ± 2.4	0.356
Carbohydrates (% EI)	41.0 ± 5.1	40.9 ± 5.0	41.1 ± 5.2	0.399
Lipids (% EI) #	41.8 ± 4.8	41.9 ± 4.7	41.8 ± 4.9	0.454
**HEI-2015 (total score) #**	59.2 ± 8.5	60.3 ± 8.5	57.9 ± 8.4	<0.001
Total Fruits (score)	3.7 ± 1.5	3.8 ± 1.4	3.6 ± 1.5	0.013
Whole Fruits (score)	3.9 ± 1.5	4.0 ± 1.5	3.8 ± 1.6	0.016
Total Vegetables (score)	3.1 ± 1.4	3.2 ± 1.4	2.9 ± 1.3	<0.001
Greens and Beans (score)	3.8 ± 1.7	3.94 ± 1.6	3.7 ± 1.8	0.087
Whole Grains (score)	1.1 ± 1.8	1.1 ± 1.7	1.2 ± 1.8	0.307
Dairy (score)	7.0 ± 2.1	6.9 ± 2.1	7.1 ± 2.0	0.224
Total Protein Foods (score)	4.9 ± 0.4	4.9 ± 0.3	4.9 ± 0.4	0.469
Seafood and Plant Proteins (score)	1.9 ± 1.4	2.0 ± 1.4	1.8 ± 1.3	0.170
(PUFAs + MUFAs)/SFAs (score)	3.2 ± 2.2	3.4 ± 2.3	2.9 ± 2.0	0.001
Refined Grains (score)	6.9 ± 2.4	7.0 ± 2.4	6.8 ± 2.4	0.206
Sodium (score)	8.6 ± 2.0	8.8 ± 1.8	8.4 ± 2.1	0.018
Added Sugars (score)	8.70 ± 1.57	8.76 ± 1.55	8.63 ± 1.58	0.145
Saturated Fats (score)	2.4 ± 2.1	2.5 ± 2.2	2.2 ± 2.1	0.026
**DASH (total score)**	23.4 ± 3.7	23.9 ± 3.7	22.8 ± 3.7	<0.001
Red meat (score)	3.0 ± 1.4	3.12 ± 1.4	2.8 ± 1.4	<0.001
Sugar drinks (score)	1.79 ± 0.41	1.83 ± 0.38	1.75 ± 0.43	0.006
Sodium (score)	2.8 ± 1.5	3.0 ± 1.4	2.6 ± 1.5	<0.001
Whole Grains (score)	3.6 ± 0.8	3.6 ± 0.8	3.6 ± 0.8	0.502
Low-fat dairy (score)	3.2 ± 1.2	3.2 ± 1.2	3.2 ± 1.2	0.885
Vegetables (score)	3.0 ± 1.4	3.1 ± 1.5	2.9 ± 1.4	0.139
Seeds, nuts and legumes (score)	3.0 ± 1.4	3.0 ± 1.4	2.9 ± 1.5	0.174
Fruits (score)	3.0 ± 1.4	3.1 ± 1.4	3.0 ± 1.4	0.252
**aDASH (total score)**	23.3 ± 3.9	23.9 ± 3.8	22.7 ± 4.0	<0.001
Red meat (score)	3.0 ± 1.4	3.1 ± 1.4	2.8 ± 1.4	0.001
Sugar drinks (score)	1.80 ± 0.40	1.83 ± 0.38	1.77 ± 0.42	0.018
Sodium (score)	2.9 ± 1.5	3.0 ± 1.5	2.7 ± 1.5	0.028
Whole Grains (score)	3.6 ± 0.8	3.6 ± 0.8	3.6 ± 0.8	0.472
Low-fat dairy (score)	3.2 ± 1.2	3.2 ± 1.2	3.2 ± 1.2	0.873
Vegetables (score)	3.0 ± 1.4	3.1 ± 1.4	2.8 ± 1.4	0.001
Seeds, nuts and legumes (score)	3.0 ± 1.4	3.0 ± 1.4	2.9 ± 1.5	0.139
Fruits (score)	3.0 ± 1.4	3.1 ± 1.4	2.9 ± 1.4	0.060

HEI-2015. Healthy eating index; DASH. Dietary Approaches to Stop Hypertension; aDASH. Alternate Dietary Approaches to Stop Hypertension; MUFAs, monounsaturated fatty acids; PUFAs, polyunsaturated fatty acids; SFAs, saturated fatty acids. # Variable follow a normal distribution. For comparison of means, the Mann-Whitney U test was used if the distribution of the variables was not homogeneous, the Student’s *t*-test for homogeneous distributions, *p*-values < 0.05 were considered statistically significant.

**Table 3:** Diet quality in the schoolchildren studied according to HOMA-IR and sex.

	Total			Girls			Boys		
	HOMA-IR ≤ 3.16 (*n* = 809)	HOMA-IR > 3.16 (*n* = 45)	*p*	HOMA-IR ≤ 3.16 (*n* = 410)	HOMA-IR > 3.16 (*n* = 31)	*p*	HOMA-IR ≤ 3.16 (*n* = 399)	HOMA-IR > 3.16 (*n* = 14)	*p*
Energy intake (kcal) #	2110 ± 350	2002 ± 330	0.022	2071 ± 336	1998 ± 361	0.123	2150 ± 360	2010 ± 261	0.076
Energy expenditure (kcal)	2121 ± 372	2194 ± 445	0.429	1892 ± 266	1998 ± 237 #	0.016	2356 ± 315	2629 ± 495	0.029
EI/EE (%)	102.1 ± 22.8	94.2 ± 22.7	0.150	111.4 ± 22.7	101.4 ± 22.0	0.013	92.6 ± 18.6	78.4 ± 15.2 #	0.002
Proteins (%)	15.6 ± 2.3	15.8 ± 2.4	0.467	15.6 ± 2.3	15.6 ± 2.1	0.983	15.5 ± 2.3	16.1 ± 2.8	0.286
Carbohydrates (%)	41.0 ± 5.1	40.3 ± 5.0	0.263	40.9 ± 5.0	40.6 ± 4.4 #	0.365	41.2 ± 5.2	39.9 ± 6.3	0.237
Lipids (%) #	41.8 ± 4.8	42.1 ± 5.1	0.336	41.8 ± 4.7	42.1 ± 4.4	0.402	41.8 ± 4.8	42.3 ± 6.6	0.357
**HEI-2015 (total score)**	59.2 ± 8.5	57.5 ± 8.6	0.094	60.5 ± 8.4	57.8 ± 9.4 #	0.044	57.9 ± 8.5	56.8 ± 6.7 #	0.319
Total Fruits (score)	3.7 ± 1.4	3.6 ± 1.6	0.930	3.8 ± 1.4	3.8 ± 1.6	0.748	3.6 ± 1.5	3.2 ± 1.5	0.402
Whole Fruits (score)	3.9 ± 1.5	3.8 ± 1.7	0.855	4.0 ± 1.5	4.0 ± 1.6	0.783	3.8 ± 1.6	3.6 ± 1.7	0.703
Total Vegetables (score)	3.1 ± 1.3	2.9 ± 1.5	0.317	3.3 ± 1.3	2.7 ± 1.5	0.043	2.9 ± 1.3	3.2 ± 1.4	0.409
Greens and Beans (score)	3.8 ± 1.7	3.6 ± 1.8	0.171	4.0 ± 1.6	3.3 ± 2.0	0.039	3.7 ± 1.8	4.1 ± 1.3	0.716
Whole Grains (score)	1.1 ± 1.8	0.6 ± 0.8	0.262	1.1 ± 1.8	0.5 ± 0.7	0.122	1.2 ± 1.8	0.8 ± 1.0	0.680
Dairy (score)	7.0 ± 2.1	7.2 ± 1.9	0.609	6.9 ± 2.1	7.3 ± 2.0	0.354	7.1 ± 2.0	7.0 ± 1.8	0.790
Total Protein Foods (score)	4.9 ± 0.4	4.9 ± 0.3	0.822	4.9 ± 0.3	4.9 ± 0.2	0.866	4.9 ± 0.4	4.8 ± 0.5	0.954
Seafood and Plant Proteins (score)	1.9 ± 1.4	2.1 ± 1.5	0.559	2.0 ± 1.4	2.1 ± 1.4	0.767	1.8 ± 1.3	2.1 ± 1.7	0.685
(PUFAs + MUFAs)/SFAs (score)	3.2 ± 2.2	2.8 ± 2.3	0.247	3.5 ± 2.3	2.9 ± 2.4 #	0.088	2.9 ± 2.0	2.7 ± 2.3	0.722
Refined Grains (score)	6.9 ± 2.4	6.7 ± 2.4	0.633	7.0 ± 2.4	6.5 ± 2.5 #	0.111	6.8 ± 2.4	7.3 ± 2.3	0.367
Sodium (score)—S	8.6 ± 2.0	8.4 ± 2.2	0.716	8.8 ± 1.8	8.9 ± 1.9	0.488	8.4 ± 2.1	7.3 ± 2.4	0.052
Added Sugars (score)	8.7 ± 1.6	8.8 ± 1.6	0.595	8.7 ± 1.6	8.9 ± 1.4	0.735	8.6 ± 1.6	8.5 ± 1.9	0.802
Saturated Fats (score)	2.4 ± 2.2	2.1 ± 1.8	0.635	2.6 ± 2.2	2.2 ± 2.0 #	0.158	2.2 ± 2.1	2.1 ± 1.6	0.810
**DASH (total score)**	23.3 ± 3.7	23.6 ± 3.6	0.605	23.9 ± 3.6	23.6 ± 3.8	0.684	22.7 ± 3.7	23.6 ± 3.3	0.381
Red meat (score)	2.9 ± 1.4	3.2 ± 1.5	0.316	3.1 ± 1.4	3.2 ± 1.4	0.768	2.8 ± 1.4	3.1 ± 1.6	0.433
Sugar drinks (score)—S	1.8 ± 0.4	1.7 ± 0.4	0.292	1.8 ± 0.4	1.8 ± 0.4	0.920	1.8 ± 0.4	1.5 ± 0.5	0.024
Sodium (score)—S	2.8 ± 1.5	3.2 ± 1.6	0.098	3.0 ± 1.4	3.5 ± 1.5	0.073	2.6 ± 1.5	2.6 ± 1.7	0.886
Whole Grains (score)	3.6 ± 0.8	3.3 ± 0.6	0.035	3.6 ± 0.8	3.3 ± 0.6	0.042	3.6 ± 0.8	3.4 ± 0.6	0.484
Low-fat dairy (score)—I	3.2 ± 1.2	3.6 ± 1.1	0.034	3.2 ± 1.2	3.5 ± 1.2	0.129	3.2 ± 1.2	3.7 ± 1.1	0.115
Vegetables (score)	3.0 ± 1.4	2.8 ± 1.6	0.424	3.1 ± 1.4	2.7 ± 1.6	0.142	2.9 ± 1.4	3.1 ± 1.5	0.538
Seeds, nuts and legumes (score)	3.0 ± 1.4	3.1 ± 1.5	0.656	3.1 ± 1.4	2.8 ± 1.6	0.419	2.9 ± 1.5	3.6 ± 1.2	0.081
Fruits (score)	3.0 ± 1.4	2.8 ± 1.3	0.212	3.1 ± 1.4	2.8 ± 1.4	0.337	3.0 ± 1.4	2.6 ± 1.3	0.324
**aDASH (total score)**	23.3 ± 3.9	23.5 ± 3.8	0.996	23.9 ± 3.8	23.7 ± 3.8	0.480	22.7 ± 4.0	23.0 ± 3.8	0.760
Red meat (score)	2.9 ± 1.4	3.1 ± 1.5	0.499	3.1 ± 1.4	3.2 ± 1.4	0.827	2.8 ± 1.4	2.9 ± 1.6	0.726
Sugar drinks (score)—S, R	1.8 ± 0.4	1.8 ± 0.4	0.408	1.8 ± 0.4	1.9 ± 0.3	0.571	1.8 ± 0.4	1.5 ± 0.5	0.015
Sodium (score)—S	2.8 ± 1.5	2.8 ± 1.5	0.969	2.9 ± 1.4	3.1 ± 1.5	0.477	2.7 ± 1.5	2.2 ± 1.4	0.176
Whole Grains (score)	3.6 ± 0.8	3.4 ± 0.6	0.073	3.6 ± 0.8	3.3 ± 0.6	0.097	3.6 ± 0.8	3.4 ± 0.6	0.508
Low-fat dairy (score)—I	3.2 ± 1.2	3.6 ± 1.1	0.028	3.2 ± 1.2	3.5 ± 1.2	0.095	3.2 ± 1.2	3.6 ± 1.1	0.148
Vegetables (score)	3.0 ± 1.4	2.9 ± 1.5	0.663	3.2 ± 1.4	2.8 ± 1.5	0.162	2.8 ± 1.4	3.1 ± 1.5	0.387
Seeds, nuts and legumes (score)	3.0 ± 1.4	3.1 ± 1.5	0.517	3.1 ± 1.4	2.9 ± 1.6	0.571	2.9 ± 1.5	3.6 ± 1.2	0.079
Fruits (score)	3.0 ± 1.4	2.8 ± 1.4	0.431	3.1 ± 1.4	3.0 ± 1.4	0.612	2.9 ± 1.4	2.6 ± 1.3	0.405

HEI-2015. Healthy eating index; DASH. Dietary Approaches to Stop Hypertension; aDASH. Alternate Dietary Approaches to Stop Hypertension MUFAs, monounsaturated fatty acids; PUFAs, polyunsaturated fatty acids; SFAs, saturated fatty acids. Two-way ANOVA analysis: S: differences according to sex; I: differences according to insulin resistance (IR) score; R: interaction between sex and IR. # Variable follow a normal distribution. For comparison of means, the Mann-Whitney U test was used if the distribution of the variables was not homogeneous, the Student’s *t*-test for homogeneous distributions, *p*-values < 0.05 were considered statistically significant.

**Table 4:** Associations between dietary indices and insulin resistance according to sex. Logistic regression analysis.

	Model 1 OR (95%CI), *p*	Model 2 OR (95%CI), *p*
**Total**		
**HEI-2015**		
T1	Ref.	Ref.
T2	0.74 (0.37–1.50) 0.406	0.67 (0.32–1.41) 0.294
T3	0.57 (0.27–1.22) 0.148	0.40 (0.17–0.95) 0.038
T2 + T3	0.66 (0.36–1.21) 0.180	0.54 (0.28–1.05) 0.071
**DASH**		
T1	Ref.	Ref.
T2	1.65 (0.79–3.44) 0.179	1.51 (0.70–3.26) 0.299
T3	1.28 (0.59–2.77) 0.531	1.09 (0.47–2.52) 0.838
T2 + T3	1.47 (0.76–2.84) 0.256	1.30 (0.65–2.62) 0.456
**aDASH**		
T1	Ref.	Ref.
T2	0.94 (0.44–2.02) 0.871	0.90 (0.41–2.00) 0.803
T3	1.14 (0.53–2.45) 0.743	1.02 (0.46–2.30) 0.955
T2 + T3	0.88 (0.47–1.62) 0.669	0.77 (0.40–1.48) 0.430
**Girls**		
**HEI-2015**		
T1	Ref.	Ref.
T2	0.46 (0.19–1.11) 0.085	0.44 (0.17–1.13) 0.086
T3	0.48 (0.20–1.17) 0.106	0.42 (0.16–1.14) 0.088
T2 + T3	0.47 (0.22–0.98) <0.001	0.43 (0.19–0.96) 0.040
**DASH**		
T1	Ref.	Ref.
T2	1.29 (0.54–3.09) 0.567	1.42 (0.57–3.55) 0.451
T3	0.96 (0.38–2.44) 0.932	0.83 (0.29–2.37) 0.733
T2 + T3	1.13 (0.52–2.45) 0.768	1.14 (0.49–2.62) 0.766
**aDASH**		
T1	Ref.	Ref.
T2	0.57 (0.22–1.45) 0.236	0.56 (0.20–1.55) 0.263
T3	0.70 (0.30–1.62) 0.510	0.70 (0.28–1.75) 0.447
T2 + T3	0.64 (0.31–1.33) 0.229	0.63 (0.29–1.41) 0.264
**Boys**		
**HEI-2015**		
T1	1.82 (0.52–6.35) 0.350	1.32 (0.35–4.98) 0.683
T2	0.81 (0.18–3.68) 0.784	0.32 (0.05–2.14) 0.242
T3	1.32 (0.41–4.29) 0.642	0.83 (0.24–2.92) 0.777
T2 + T3	1.82 (0.52–6.35) 0.350	1.32 (0.35–4.98) 0.683
**DASH**		
T1	Ref.	Ref.
T2	2.65 (0.65–10.81) 0.174	1.79 (0.39–8.11) 0.453
T3	2.21 (0.22–9.42) 0.285	2.44 (0.54–10.99) 0.246
T2 + T3	2.43 (0.67–8.84) 0.178	2.07 (0.54–7.97) 0.289
**aDASH**		
T1	Ref.	Ref.
T2	1.75 (0.46–6.67) 0.660	1.07 (0.24–4.76) 0.927
T3	1.51 (0.40–5.74) 0.558	1.47 (0.37–5.89) 0.584
T2 + T3	1.62 (0.50–5.26) 0.421	1.27 (0.37–4.43) 0.703

Model 1. Crude model. Model 2. Adjusted for age, sex, z-score of BMI, and activity coefficient. HEI-2015. Healthy eating index; DASH. Dietary Approaches to Stop Hypertension; aDASH. Alternate Dietary Approaches to Stop Hypertension, *p*-values < 0.05 were considered statistically significant.

## Data Availability

The data presented in this study are available on request from the corresponding author.

## Supplementary Materials

The following supporting information can be downloaded at: https://www.mdpi.com/article/10.3390/nu14204232/s1, Table S1. Anthropometric, physical activity and blood biochemical parameters in the schoolchildren studied according to sex and age. Table S2. Anthropometric, physical activity and blood biochemical parameters in the schoolchildren studied according to sex and HOMA-IR. Table S3. Parameters of different index in the schoolchildren studied according to sex. Table S4. Diet quality in the schoolchildren studied according to sex and age. Table S5. Parameters of different index in the schoolchildren studied according to HOMA-IR and sex. Table S6. Diet quality in the schoolchildren studied according to HOMA-IR and age. Table S7. Tertiles of the different indices according to sex.
