# Supplementary material for: Association of Healthy Eating Index-2015 and Dietary Approaches to Stop Hypertension Patterns with Insulin Resistance in Schoolchildren
Source: Nutrients. 2022 Oct 11;14(20):4232. doi: 10.3390/nu14204232 (PMC9607022; doi:10.3390/nu14204232)
Supplement: Supplementary file 1 [file nutrients-14-04232-s001.zip › nutrients-1929136-supplementary/nutrients-1929136-supplementary/Table S1. Anthropometric, physical activity and blood biochemical parameters in the schoolchildr.pdf]

Table S1. Anthropometric, physical activity and blood biochemical parameters in the schoolchildren studied according to sex and age.

	Total			Girls			Boys		
	**08-10 years (n=541)**	**11-13 years (n=313)**	**p**	**08-10 years (n=272)**	**11-13 years (n=169)**	**p**	**08-10 years (n=269)**	**11-13 years (n=144)**	**p**
Madrid [%(n)]	53.2(288)	59.7(187)	**0.004**	56.3(153)	63.3(107)	0.268	50.2(135)	55.6(80)	**0.025**
Barcelona [%(n)]	6.8(37)	9.6(30)		6.6(18)	8.3(14)		7.1(19)	11.1(16)	
Sevilla [%(n)]	12.6(68)	12.1(38)		12.1(33)	10.7(18)		13.0(35)	13.9(20)	
A Coruña [%(n)]	13.7(74)	12.8(40)		11.8(32)	10.7(18)		15.6(42)	15.3(22)	
Valencia [%(n)]	13.7(74)	5.8(18)		13.2(36)	7.1(12)		14.1(38)	4.2(6)	
**Body composition**									
Weight (kg) -A	**36.7±8.2**	**44.1±9.4**	**<0.001**	**36.4±7.4**	**44.8±9.4**	**<0.001**	**37.1±8.8**	**43.2±9.4**	**<0.001**
Height (m) # -SA	**1.4±0.1**	**1.5±0.1**	**<0.001**	**1.4±0.1**	**1.5±0.1**	**<0.001**	**1.4±0.1**	**1.5±0.1**	**<0.001**
BMI (kg/m2) -A	**18.6±3.1**	**19.7±3.0**	**<0.001**	**18.4±2.9**	**19.7±2.9**	**<0.001**	**18.8±3.4**	**19.6±3.1**	**0.003**
Z-BMI #-S	0.68±1.19	0.69±1.02	0,498	0.54±1.07	0.62±0.99	0.216	0.83±1.28	0.76±1.05	0.276
**Nutritional status by BMI**									
Underweight [%(n)]	0.7(4)	1.0(3)	0.386	0.7(2)	0.6(1)	0.482	0.7(2)	1.4(2)	0.540
Normal weight [%(n)]	58.8(318)	60.4(189)		64.3(175)	61.5(104)		53.2(143)	59.0(85)	
Overweight [%(n)]	26.6(144)	28.8(90)		26.8(73)	32.5(55)		26.4(71)	24.3(35)	
Obesity [%(n)]	13.9(75)	9.9(31)		8.1(22)	5.3(9)		19.7(53)	15.3(22)	
Body fat (%) # -SA	**27.0±6.1**	**28.7±4.7**	**<0.001**	**28.7±5.0**	**30.3±3.8**	**<0.001**	**25.2±6.5**	**26.9±5.0**	**0.002**
**Nutritional status by body fat percentage**									
Low fat [%(n)]	0.6(3)	0.0(0)	**<0.001**	0.4(1)	0.0(0)	**0.046**	0.7(2)	0.0(0)	**0.002**
Normal fat [%(n)]	40.4(218)	30.0(94)		43.8(119)	34.9(59)		37.1(99)	24.3(35)	
Fat excessive [%(n)]	26.2(141)	40.9(128)		28.3(77)	40.8(69)		24.0(64)	41.0(59)	
Obesity [%(n)]	32.8(177)	29.1(91)		27.6(75)	24.3(41)		38.2(102)	34.7(50)	
**Physical activity**									
Activity coefficient -A	**1.54±0.11**	**1.51±0.11**	**0.007**	1.53±0.11	1.51±0.10	0.089	1.54±0.10	1.52±0.12	**0.045**
**Biochemical data**									
Glucose (mg/dL) -S	84.0±10.0	85.1±9.0	0.112	82.7±10.2	84.5±9.6	0.134	85.3±9.7	85.9±8.2	0.393
Insulin (mcU/mL) -SA	**5.8±4.0**	**7.3±4.8**	**<0.001**	**6.3±4.3**	**8.3±5.3**	**<0.001**	**5.2±3.5**	**6.1±3.9**	**0.005**
QUICKI -SA	**0.39±0.05**	**0.37±0.04**	**<0.001**	**0.39±0.05**	**0.37±0.04**	**<0.001**	**0.40±0.04**	**0.38±0.04**	**0.004**
HOMA-IR -SA	**1.21±0.87**	**1.54±1.09**	**<0.001**	**1.31±0.94**	**1.74±1.23**	**<0.001**	**1.10±0.77**	**1.30±0.86**	**0.004**
IR [%(n)] -SA	**3.51(19)**	**8.31(26)**	**0.003**	**4.78(13)**	**10.65(18)**	**0.023**	**2.23(6)**	**5.56(8)**	**0.044**

BMI, body mass index; IR, insulin resistance. Two-way ANOVA analysis: S: differences according to sex; A: differences according to age; R: interaction between sex and age. # Variable follow a normal distribution. For comparison of means, the Mann-Whitney U test was used if the distribution of the variables was not homogeneous, the Student's t-test for homogeneous distributions.
