# Supplementary material for: Association of Healthy Eating Index-2015 and Dietary Approaches to Stop Hypertension Patterns with Insulin Resistance in Schoolchildren
Source: Nutrients. 2022 Oct 11;14(20):4232. doi: 10.3390/nu14204232 (PMC9607022; doi:10.3390/nu14204232)
Supplement: Supplementary file 1 [file nutrients-14-04232-s001.zip › nutrients-1929136-supplementary/nutrients-1929136-supplementary/Table S2. Anthropometric, physical activity and blood biochemical parameters in the schoolchildr.pdf]

Table S2. Anthropometric, physical activity and blood biochemical parameters in the schoolchildren studied according to sex and HOMA-IR

	Total			Girls			Boys		
	HOMA-IR≤3.16 (n=809)	HOMA-IR>3.16 (n=45)	p	HOMA-IR≤3.16 (n=410)	HOMA-IR>3.16 (n=31)	p	HOMA-IR≤3.16 (n=399)	HOMA-IR>3.16 (n=14)	p
Age (years) – I	**10.1±0.9**	**10.5±1.0**	**0.003**	**10.1±0.9**	**10.5±1.0**	**0.039**	**10.1±0.9**	**10.6±1.0**	0.035
Madrid [%(n)]	56.1(454)	46.7(21)	0.190	61.0(250)	32.3(10)	**0.002**	51(204.0)	79(11.0)	0.272
Barcelona [%(n)]	7.5(61)	13.3(6)		6.3(26)	19.4(6)		9(35.0)	0(0.0)	
Sevilla [%(n)]	12.5(101)	11.1(5)		11.5(47)	12.9(4)		14(54.0)	7(1.0)	
A Coruña [%(n)]	12.9(104)	22.2(10)		10.2(42)	25.8(8)		16(62.0)	14(2.0)	
Valencia [%(n)]	11.0(89)	6.7(3)		11.0(45)	9.7(3)		11(44.0)	0(0.0)	
**Body composition**									
Weight (kg) – I	**38.9±9.0**	**48.4±10.6**	**<0.001**	**39.0±9.0**	**47.0±8.8**	**<0.001**	**38.8±9.0**	**51.3±13.8**	**<0.001**
Height (m) # – I	**1.4±0.1**	**1.5±0.1**	**<0.001**	**1.4±0.1**	**1.5±0.1**	**<0.001**	**1.4±0.1**	**1.5±0.1**	0.044
BMI (kg/m2) – SIR	**18.8±3.0**	**21.8±3.9**	**<0.001**	**18.7±2.9**	**21.0±2.8**	**<0.001**	**18.9±3.1**	**23.8±5.1**	**<0.001**
Z-BMI – SI	**0.64±1.11**	**1.50±1.12#**	**<0.001**	**0.52±1.04**	**1.21±0.81**	**<0.001**	**0.76±1.17**	**2.13±1.46#**	**<0.001**
**Nutritional status by BMI**									
Underweight [%(n)]	0.9(7)	0.0(0)	**<0.001**	0.7(3)	0.0(0)	**0.002**	1(4.0)	0(0.0)	**<0.001**
Normal weight [%(n)]	61.1(494)	28.9(13)		65.6(269)	32.3(10)		56(225.0)	21(3.0)	
Overweight [%(n)]	26.7(216)	40.0(18)		27.3(112)	51.6(16)		26(104.0)	14(2.0)	
Obesity [%(n)]	11.4(92)	31.1(14)		6.3(26)	16.1(5)		17(66.0)	64(9.0)	
Body fat (%) # – SI	**27.4±5.7**	**31.6±3.9**	**<0.001**	**29.1±4.7**	**31.8±3.4**	**<0.001**	**25.6±6.0**	**31.1±4.8**	**<0.001**
**Nutritional status by body fat percentage**									
Low fat [%(n)]	0.4(3)	0.0(0)	**0.003**	0.2(1)	0.0(0)	**0.009**	1(2.0)	0(0.0)	**0.047**
Normal fat [%(n)]	38.0(307)	11.1(5)		42.4(174)	12.9(4)		34(133.0)	7(1.0)	
Fat excessive [%(n)]	30.9(249)	44.4(20)		31.5(129)	54.8(17)		30(120.0)	21(3.0)	
Obesity [%(n)]	30.7(248)	44.4(20)		25.9(106)	32.3(10)		36(142.0)	71(10.0)	
**Physical activity**									
Activity coefficient– S	**1.53±0.11**	**1.49±0.10**	**0.008**	**1.53±0.11**	**1.47±0.07#**	**<0.001**	1.53±0.11	1.54±0.13#	0.449
**Biochemical data**									
Glucose (mg/dL)	**84.2±9.6**	**89.0±10.4**	**0.002**	**83.0±10.0**	**88.5±8.5**	**<0.001**	85.3±9.0	90.1±14.0	0.511
Insulin (mcU/mL) – SI	**5.6±3.1**	**18.9±5.0**	**<0.001**	**6.1±3.2**	**19.3±5.6**	**<0.001**	**5.1±2.8**	**18.0±3.4**	**<0.001**
QUICKI – I	**0.39±0.04**	**0.31±0.01**	**<0.001**	**0.38±0.04**	**0.31±0.01**	**<0.001**	**0.39±0.04**	**0.31±0.01**	**<0.001**
HOMA-IR – I	**1.17±0.66**	**4.13±1.29**	**<0.001**	**1.27±0.69**	**4.23±1.48**	**<0.001**	**1.07±0.61**	**3.93±0.68**	**<0.001**

BMI, body mass index; IR, insulin resistance .Two-way ANOVA analysis: S: differences according to sex; I: differences according to insulin resistance (IR) score; R: interaction between sex and IR. # Variable follow a normal distribution. For comparison of means, the Mann-Whitney U test was used if the distribution of the variables was not homogeneous, the Student's t-test for homogeneous distributions.
