# Supplementary material for: Association of Healthy Eating Index-2015 and Dietary Approaches to Stop Hypertension Patterns with Insulin Resistance in Schoolchildren
Source: Nutrients. 2022 Oct 11;14(20):4232. doi: 10.3390/nu14204232 (PMC9607022; doi:10.3390/nu14204232)
Supplement: Supplementary file 1 [file nutrients-14-04232-s001.zip › nutrients-1929136-supplementary/nutrients-1929136-supplementary/Table S3. Parameters of different index in the schoolchildren studied according to sex.pdf]

Table S3. Parameters of different index in the schoolchildren studied according to sex

	**Total (n=854)**	**Girls (n=441)**	**Boys (n=413)**	**p**
**HEI-2015**				
Total Fruits (cups/1000kcal)	0.71±0.40	**0.74±0.40**	**0.69±0.40**	**0.029**
Whole Fruits (cups/1000kcal)	0.47±0.32	0.49±0.32	0.46±0.32	0.070
Total Vegetables (cups/1000kcal)	0.74±0.46	**0.80±0.50**	**0.69±0.40**	**<0.001**
Greens and Beans (cups/1000kcal)	0.34±0.35	0.36±0.39	0.31±0.31	0.075
Whole Grains (oz/1000kcal)	0.17±0.27	0.16±0.27	0.17±0.28	0.308
Dairy (cups/1000kcal)	0.93±0.31	0.92±0.32	0.95±0.31	0.248
Total Protein Foods (oz/1000kcal)	3.6±1.1	3.6±1.08	3.6±1.0	0.883
Seafood and Plant Proteins (cups/1000kcal)	0.71±0.57	0.74±0.58	0.68±0.55	0.176
(PUFAs+MUFAs)/SFAs	1.61±0.30	**1.64±0.32**	**1.57±0.28**	**<0.001**
Refined Grains (oz/1000kcal) #	2.55±0.67	2.52±0.69	2.58±0.65	0.100
Sodium (mg/1000kcal)	1169±239	1153±220	1187±257	0.063
Added Sugars (% of kcal)	8.5±3.6	8.3±3.6	8.7±3.6	0.096
Saturated Fats (% of kcal) #	14.4±2.1	**14.2±2.1**	**14.6±2.2**	**0.009**
**DASH**				
Red meat (g)	104.8±49.7	**98.0±48.3**	**112.0±50.3**	**<0.001**
Sugar drinks (g)	51.8±93.1	**44.2±83.9**	**59.9±101.5**	**0.027**
Sodium (mg)	2451±611	**2372±566**	**2536±645**	**<0.001**
Whole Grains (g)	9.9±16.4	9.4±16.1	10.5±16.6	0.223
Low-fat dairy (g)	98.3±129.0	96.4±122.7	100.4±135.5	0.846
Vegetables (g)	124.8±59.8	129.2±62.9	120.1±55.9	0.065
Seeds, nuts and legumes (g)	28.2±29.8	28.8±30.0	27.5±29.6	0.326
Fruits (g)	270.1±161.0	273.9±157.0	266.0±165.4	0.243
**aDASH**				
Red meat (g/1000kcal)	50.1±23.2	**47.8±23.1**	**52.6±23.1**	**0.002**
Sugar drinks (g/1000kcal)	24.6±45.3	**21.4±41.9**	**28.1±48.5**	**0.035**
Sodium (mg/1000kcal)	1169±239	1153±220	1187±257	0.063
Whole Grains (g/1000kcal)	4.7±7.6	4.5±7.5	4.9±7.7	0.314
Low-fat dairy (g/1000kcal)	47.6±63.0	47.6±62.1	47.6±64.0	0.991
Vegetables (g/1000kcal)	59.7±27.9	**62.6±28.7**	**56.6±26.7**	**0.002**
Seeds, nuts and legumes (g/1000kcal)	13.4±14.1	13.8±14.1	13.0±14.2	0.194
Fruits (g/1000kcal)	129.1±75.4	133.7±76.5	124.2±74.0	0.065

HEI-2015. Healthy eating index; DASH. Dietary Approaches to Stop Hypertension; aDASH. Alternate Dietary Approaches to Stop Hypertension. MUFAs, monounsaturated fatty acids; PUFAs, polyunsaturated fatty acids; SFAs, saturated fatty acids. # Variable follow a normal distribution. For comparison of means, the Mann-Whitney U test was used if the distribution of the variables was not homogeneous, the Student's t-test for homogeneous distributions.
