# Supplementary material for: Association of Healthy Eating Index-2015 and Dietary Approaches to Stop Hypertension Patterns with Insulin Resistance in Schoolchildren
Source: Nutrients. 2022 Oct 11;14(20):4232. doi: 10.3390/nu14204232 (PMC9607022; doi:10.3390/nu14204232)
Supplement: Supplementary file 1 [file nutrients-14-04232-s001.zip › nutrients-1929136-supplementary/nutrients-1929136-supplementary/Table S4. Diet quality in the schoolchildren studied according to sex and age..pdf]

Table S4. Diet quality in the schoolchildren studied according to sex and age.

	Total			Girls			Boys		
	**8-10 years**	**11-13 years**	**P**	**8-10 years**	**11-13 years**	**P**	**8-10 years**	**11-13 years**	**P**
Energy intake (EI) (kcal) -S	2103±347	2108±355#	0.421	2068±350	2063±318	0.939	2138±341	2160±388#	0.276
Energy expenditure (EE) (kcal) -SA	**2085±364**	**2193±389**	**<0.001**	**1845±241**	**1986±280**	**<0.001**	**2328±301**	**2436±358**	**0.004**
EE/EI (%) -SA	**103.6±23.5**	**98.5±21.4#**	**<0.001**	**113.9±23.6**	**105.6±20.4**	**<0.001**	93.2±18.1	90.2±19.6#	0.061
Proteins (%)	15.5±2.3	15.8±2.4	0.120	15.5±2.2	15.8±2.5	0.531	15.4±2.4	15.7±2.3	0.128
Carbohydrates (%)	41.0±5.1	40.9±5.0	0.796	40.9±5.2	40.8±4.6	0.834	41.2±5.1	41.0±5.5	0.351
Lipids (%) #	41.9±4.7	41.8±4.8	0.417	41.9±4.7	41.8±4.6	0.413	41.8±4.8	41.8±5.1	0.467
**HEI-2015 (total score) # -S**	**59.5±8.3**	**58.5±8.9**	**0.039**	**61.0±8.1**	**59.3±8.9**	**0.024**	58.1±8.2	57.5±8.8	0.235
Total Fruits (score) -S	3.7±1.4	3.7±1.5	0.821	3.9±1.4	3.7±1.5	0.711	3.6±1.4	3.5±1.6	0.940
Whole Fruits (score) -S	4.0±1.5	3.8±1.6	0.404	4.1±1.5	4.0±1.5	0.569	3.9±1.5	3.7±1.8	0.436
Total Vegetables (score) -S	3.1±1.4	3.1±1.3	0.774	3.3±1.4	3.1±1.3	0.182	2.9±1.4	3.0±1.3	0.353
Greens and Beans (score) -S	3.8±1.7	3.8±1.7	0.661	3.9±1.6	3.9±1.6	0.604	3.7±1.7	3.6±1.8	0.824
Whole Grains (score)	1.1±1.7	1.1±1.8	0.383	1.1±1.8	1.0±1.7	0.391	1.1±1.7	1.2±2.0	0.745
Dairy (score) -A	**7.1±2.1**	**6.8±2.1**	**0.011**	7.0±2.1	6.7±2.1	0.123	**7.2±2.0**	**6.8±2.1**	**0.045**
Total Protein Foods (score)	4.9±0.4	4.9±0.3	0.085	4.9±0.4	4.9±0.3	0.404	4.9±0.4	4.9±0.3	0.117
Seafood and Plant Proteins (score)	1.9±1.4	1.9±1.4	0.629	2.0±1.4	2.0±1.4	0.881	1.8±1.3	1.9±1.4	0.411
(PUFAs+MUFAs)/SFAs (score) -S	3.2±2.2	3.2±2.3	0.964	3.5±2.3	3.4±2.4	0.502	2.9±2.0	3.0±2.1	0.620
Refined Grains (score)	7.0±2.4	6.7±2.4	0.068	**7.1±2.4**	**6.7±2.4**	**0.037**	6.8±2.4	6.7±2.4	0.598
Sodium (score) -SA	**8.7±1.8**	**8.3±2.2**	**0.038**	8.9±1.6	8.5±2.0	0.111	8.6±1.9	8.1±2.4	0.135
Added Sugars (score)	8.6±1.6	8.8±1.5	0.164	8.7±1.6	8.8±1.5	0.412	8.6±1.6	8.7±1.6	0.294
Saturated Fats (score) -S	2.3±2.1	2.4±2.2	0.657	2.5±2.1	2.5±2.3	0.827	2.1±2.1	2.3±2.1	0.412
Total Fruits (cups/1000kcal)	**0.71±0.40**	**0.71±0.41**	**0.029**	0.74±0.39	0.73±0.40	0.701	0.68±0.40	0.69±0.42	0.881
Whole Fruits (cups/1000kcal)	**0.48±0.32**	**0.46±0.32**	**0.070**	0.49±0.32	0.48±0.32	0.656	0.47±0.32	0.44±0.32	0.471
Total Vegetables (cups/1000kcal) -S	**0.75±0.46**	**0.74±0.44**	**0.000**	0.82±0.51	0.76±0.47	0.172	0.67±0.39	0.71±0.41	0.339
Greens and Beans (cups/1000kcal)	**0.34±0.36**	**0.33±0.34**	**0.075**	0.38±0.41	0.33±0.35	0.424	0.30±0.30	0.32±0.33	0.875
Whole Grains (oz/1000kcal)	0.17±0.26	0.16±0.28	0.308	0.17±0.27	0.14±0.26	0.393	0.17±0.26	0.18±0.31	0.747
Dairy (cups/1000kcal) -A	0.95±0.31	0.90±0.32	0.248	0.94±0.32	0.90±0.32	0.135	**0.96±0.30**	**0.91±0.32#**	**0.043**
Total Protein Foods (oz/1000kcal)	3.5±1.0	3.7±1.1	0.883	3.6±1.1	3.6±1.1	0.672	**3.5±1.0**	**3.8±1.1#**	**0.003**
Seafood and Plant Proteins (cups/1000kcal) -A	0.70±0.54	0.73±0.61	0.176	0.74±0.57	0.74±0.61	0.878	0.66±0.52	0.73±0.61	0.410
(PUFAs+MUFAs)/SFAs	**1.61±0.30**	**1.60±0.31**	**0.000**	1.65±0.31	1.63±0.33 #	0.229	1.57±0.28	1.58±0.28#	0.394
Refined Grains (oz/1000kcal)# -S	2.5±0.7	2.6±0.7	0.100	**2.5±0.7**	**2.6±0.7**	**0.025**	2.6±0.6	2.6±0.7	0.360
Sodium (mg/1000kcal) -SA	1152±220	1200±266	0.063	1137±200	1178±248	0.141	1166±239	1225±286	0.082
Added Sugars (% of kcal)	8.7±3.6	8.3±3.6	0.096	8.5±3.7	8.1±3.5	0.442	8.9±3.5	8.4±3.7	0.123
Saturated Fats (% of kcal) #	**14.4±2.1**	**14.4±2.2**	**0.009**	14.2±2.0	14.3±2.2	0.332	14.6±2.1	14.5±2.2	0.226
**DASH (total score)**	**23.6±3.7**	**23.0±3.8**	**0.048**	24.2±3.6	23.4±3.7	0.019	22.9±3.6	22.5±3.9	0.468
Red meat (score)	**3.0±1.4**	**2.8±1.4**	**0.041**	3.1±1.4	3.1±1.4	0.922	**2.9±1.4**	**2.5±1.4**	**0.002**
Sugar drinks (score)	**1.82±0.39**	**1.76±0.43**	**0.037**	**1.86±0.35**	**1.79±0.41**	**0.045**	1.77±0.42	1.72±0.45	0.251
Sodium (score)	2.9±1.5	2.7±1.5	0.073	3.1±1.4	2.9±1.4	0.264	2.7±1.5	2.5±1.5	0.082
Whole Grains (score)	3.6±0.8	3.5±0.8	0.237	3.6±0.8	3.5±0.7	0.119	3.6±0.8	3.6±0.8	0.973
Low-fat dairy (score)	3.2±1.2	3.2±1.2	0.656	3.2±1.2	3.2±1.2	0.681	3.2±1.2	3.3±1.2	0.279
Vegetables (score)	3.0±1.4	3.0±1.4	0.853	3.1±1.4	3.0±1.5	0.641	2.9±1.4	3.0±1.3	0.461

	Total			Girls			Boys		
	**8-10 years**	**11-13 years**	**P**	**8-10 years**	**11-13 years**	**P**	**8-10 years**	**11-13 years**	**P**
Seeds. nuts and legumes (score)	3.0±1.4	2.9±1.5	0.146	**3.2±1.4**	**2.8±1.4**	**0.020**	2.9±1.4	2.9±1.6	0.861
Fruits (score)	3.0±1.4	3.0±1.4	0.902	3.1±1.4	3.0±1.4	0.703	2.9±1.4	3.0±1.4	0.581
Red meat (g) -SA	**101.4±46.8**	**110.6±53.9**	**<0.001**	96.4±43.9	100.5±54.6	0.879	**106.4±49.1**	**122.5±50.8**	**0.001**
Sugar drinks (g) -S	**49.4±89.0**	**56.0±99.7**	**0.027**	42.7±84.0	46.8±83.8	0.908	56.1±93.5	66.9±115.1	0.549
Sodium (mg) -S	**2412±573**	**2519±668**	**<0.001**	2338±521	2425±630	0.210	2487±612	2628±696	0.083
Whole Grains (g)	10.0±15.8	9.7±17.3	0.223	9.9±16.3	8.6±15.9	0.310	10.2±15.3	11.0±18.8	0.759
Low-fat dairy (g)	97.9±130.8	99.0±125.8	0.846	100.8±129.3	89.3±111.2	0.794	95.0±132.5	110.4±140.6	0.264
Vegetables (g)	124.4±61.5	125.5±56.7	0.065	131.2±65.2	125.9±58.9	0.617	117.5±56.7	125.1±54.2	0.241
Seeds. nuts and legumes (g)	28.8±29.4	27.1±30.6	0.326	**30.4±29.2**	**26.2±31.1**	**0.014**	27.1±29.5	28.2±29.9	0.881
Fruits (g)	269.5±157.7	271.1±166.9	0.243	275.8±154.9	270.8±160.6	0.628	263.1±160.5	271.4±174.5	0.717
**aDASH (total score)** -SA	23.5±3.9	23.0±3.9	0.059	**24.3±3.7**	**23.4±3.9**	**0.016**	22.8±4.0	22.5±4.0	0.632
Red meat (score) -SA	**3.0±1.4**	**2.8±1.4**	**0.038**	3.1±1.4	3.1±1.4	0.698	**2.9±1.4**	**2.5±1.3**	**0.005**
Sugar drinks (score) -S	1.8±0.4	1.8±0.4	0.132	1.9±0.4	1.8±0.4	0.186	1.8±0.4	1.7±0.4	0.342
Sodium (score) -SA	**2.9±1.5**	**2.7±1.5**	**0.013**	3.0±1.4	2.8±1.5	0.094	**2.8±1.5**	**2.6±1.5**	**0.047**
Whole Grains (score)	3.6±0.8	3.6±0.8	0.512	3.6±0.8	3.5±0.8	0.297	3.6±0.8	3.6±0.8	0.850
Low-fat dairy (score)	3.2±1.2	3.3±1.2	0.490	3.2±1.2	3.2±1.2	0.781	3.1±1.2	3.3±1.2	0.202
Vegetables (score) -S	3.0±1.4	3.0±1.4	0.728	3.2±1.4	3.1±1.5	0.530	2.8±1.4	2.9±1.4	0.265
Seeds. nuts and legumes (score)	3.0±1.4	2.9±1.5	0.104	**3.2±1.3**	**2.8±1.4**	**0.013**	2.9±1.4	2.9±1.5	0.934
Fruits (score)	3.0±1.4	3.0±1.5	0.846	3.1±1.4	3.1±1.5	0.791	2.9±1.4	3.0±1.4	0.645
Red meat (g/1000kcal) -SA	**48.5±22.0**	**52.9±24.9**	**0.002**	47.0±21.6	49.0±25.3	0.649	**50.0±22.3**	**57.5±23.8**	**0.002**
Sugar drinks (g/1000kcal)-S	**23.4±43.7**	**26.7±47.9**	**0.035**	20.5±41.5	22.7±42.5	0.907	26.2±45.6	31.4±53.4	0.514
Sodium (mg/1000kcal) -SA	1152±220	1200±266	0.063	1137±200	1178±248	0.141	1166±239	1225±286	0.082
Whole Grains (g/1000kcal)	1.2±0.2	1.2±0.3	0.314	1.1±0.2	1.2±0.2	0.315	1.2±0.2	1.2±0.3	0.743
Low-fat dairy (g/1000kcal)	47.7±64.5	47.4±60.3	0.991	49.9±65.4	44.0±56.4	0.788	45.5±63.7	51.4±64.5	0.280
Vegetables (g/1000kcal) -S	**59.2±27.8**	**60.5±28.0**	**0.002**	63.1±28.4	61.6±29.3	0.498	55.2±26.6	59.1±26.6	0.180
Seeds. nuts and legumes (g/1000kcal)	13.7±14.0	12.9±14.3	0.194	**14.6±13.8**	**12.5±14.4**	**0.014**	12.9±14.1	13.3±14.3	0.777
Fruits (g/1000kcal)	128.8±73.6	129.6±78.5	0.065	134.6±75.3	132.2±78.6	0.678	122.9±71.5	126.6±78.5	0.742

HEI-2015. Healthy eating index; DASH. Dietary Approaches to Stop Hypertension; aDASH. Alternate Dietary Approaches to Stop Hypertension MUFAs, monounsaturated fatty acids; PUFAs, polyunsaturated fatty acids; SFAs, saturated fatty acids. Two-way ANOVA analysis: S: differences according to sex; A: differences according to age; R: interaction between sex and IR. # Variable follow a normal distribution. For comparison of means, the Mann-Whitney U test was used if the distribution of the variables was not homogeneous, the Student's t-test for homogeneous distributions.
