# Supplementary material for: Association of Healthy Eating Index-2015 and Dietary Approaches to Stop Hypertension Patterns with Insulin Resistance in Schoolchildren
Source: Nutrients. 2022 Oct 11;14(20):4232. doi: 10.3390/nu14204232 (PMC9607022; doi:10.3390/nu14204232)
Supplement: Supplementary file 1 [file nutrients-14-04232-s001.zip › nutrients-1929136-supplementary/nutrients-1929136-supplementary/Table S5. Parameters of different index in the schoolchildren studied according to HOMA-IR and sex.pdf]

Table S5. Parameters of different index in the schoolchildren studied according to HOMA-IR and sex

	**Total**			**Girls**			**Boys**		
	**HOMA-IR ≤3.16 (n=809)**	**HOMA-IR >3.16 (n=45)**	**p**	**HOMA-IR ≤3.16 (n=410)**	**HOMA-IR >3.16 (n=31)**	**p**	**HOMA-IR ≤3.16 (n=399)**	**HOMA-IR >3.16 (n=14)**	**p**
**HEI-2015**									
Total Fruits (cups/1000kcal)	0.71±0.40	0.71±0.43	0.976	0.74±0.39	0.75±0.43	0.763	0.69±0.40	0.64±0.45	0.499
Whole Fruits (cups/1000kcal)	0.47±0.32	0.52±0.37	0.473	0.48±0.32	0.51±0.32	0.468	0.46±0.32	0.52±0.48	0.997
Total Vegetables (cups/1000kcal)	0.74±0.45	0.74±0.54	0.353	0.81±0.49	0.71±0.57	0.058	0.68±0.40	0.79±0.47	0.413
Greens and Beans (cups/1000kcal)	0.33±0.35	0.35±0.43	0.530	0.36±0.38	0.34±0.46	0.125	0.31±0.31	0.39±0.36	0.349
Whole Grains (oz/1000kcal)	0.17±0.28	0.09±0.13	0.262	0.16±0.27	0.07±0.11	0.122	0.18±0.28	0.12±0.15	0.681
Dairy (cups/1000kcal)	0.93±0.32	0.96±0.29	0.601	0.92±0.32	0.96±0.27	0.368	0.95±0.31	0.95±0.33 #	0.460
Total Protein Foods (oz/1000kcal)	3.6±1.1	3.7±1.1	0.635	3.6±1.1	3.5±1.0	0.652	3.6±1.0	4.0±1.3 #	0.069
Seafood and Plant Proteins (cups/1000kcal)	0.71±0.56	0.78±0.62	0.555	0.74±0.58	0.77±0.58	0.767	0.68±0.54	0.81±0.73	0.682
(PUFAs+MUFAs)/SFAs	1.6±0.3	1.6±0.3	0.272	1.6±0.3	1.6±0.3 #	0.103	1.6±0.3	1.5±0.3	0.776
Refined Grains (oz/1000kcal) #	2.5±0.7	2.6±0.6	0.292	2.5±0.7	2.7±0.6	0.113	2.6±0.6	2.5±0.6	0.244
Sodium (mg/1000kcal) - S	1169±238	1181±257	0.232	1155±219	1128±233	0.109	1183±256	1300±274	0.720
Added Sugars (% of kcal)	8.5±3.6	8.3±3.7	0.633	8.4±3.6	8.1±3.4	0.864	8.7±3.6	8.7±4.4	0.703
Saturated Fats (% of kcal)	14.4±2.1	14.7±2.2 #	0.133	14.2±2.1	14.7±2.2 #	0.097	14.6±2.1	14.8±2.4	0.327
**DASH**									
Red meat (g) - S	105.0±49.6	100.9±52.3	0.418	98.2±48.5	94.6±45.3	0.742	111.9±49.8	114.9±65.1	0.721
Sugar drinks (g) - S	51.5±93.0	56.6±96.4	0.677	44.6±84.3	39.9±78.7	0.849	58.7±100.7	93.4±122.5	0.188
Sodium (mg) - S	2457±612	2351±595	0.233	2383±571	2226±488	0.151	2533±643	2629±728	0.638
Whole Grains (g)	10.2±16.7	5.0±7.0	0.213	9.8±16.6	4.4±6.9	0.111	10.6±16.8	6.3±7.4	0.780
Low-fat dairy (g)	97.0±129.4	122.5±119.8	0.057	94.6±122.2	119.9±128.1	0.180	99.4±136.4	128.2±103.2	0.172
Vegetables (g)	125.1±59.6	118.7±63.5	0.416	130.3±62.7	113.6±64.7	0.137	119.8±55.8	130.0±61.4	0.558
Seeds, nuts and legumes (g)	28.1±29.9	29.3±28.4	0.805	29.0±30.2	25.3±26.4	0.342	27.2±29.5	38.1±31.6	0.094
Fruits (g)	271.6±161.2	243.3±156.3	0.205	275.3±156.7	255.0±161.7	0.429	267.7±165.9	217.4±145.8	0.217
**aDASH**									
Red meat (g/1000kcal)	50.1±23.0	51.0±27.0	0.745	47.8±23.0	47.9±24.2	0.913	52.5±22.8	57.7±32.4	0.962
Sugar drinks (g/1000kcal) - S	24.5±45.3	26.9±45.2	0.683	21.5±42.2	19.3±37.3	0.857	27.5±48.1	43.8±57.1	0.197
Sodium (mg/1000kcal) - S	1169±238	1181±257	0.232	1155±219	1128±233	0.109	1183±256	1300±274	0.720
Whole Grains (g/1000kcal)	4.8±7.7	2.4±3.5	0.865	4.7±7.7	2.0±3.1	0.406	4.9±7.8	3.3±4.4	0.083
Low-fat dairy (g/1000kcal)	**46.9±63.3**	**59.5±56.6**	**0.044**	46.8±62.3	58.2±59.5	0.141	47.0±64.4	62.4±51.3	0.164
Vegetables (g/1000kcal)	59.7±27.6	59.7±32.6	0.729	62.9±28.3	57.8±34.0	0.214	56.3±26.5	64.1±30.0	0.390
Seeds, nuts and legumes (g/1000kcal)	13.3±14.1	14.7±14.0	0.551	13.9±14.1	12.8±13.2	0.485	12.8±14.1	19.1±15.4	0.058
Fruits (g/1000kcal)	129.5±75.4	121.2±75.0	0.457	134.1±76.3	128.0±79.2	0.756	124.8±74.3	106.4±64.9	0.338

HEI-2015. Healthy index; DASH. Dietary Approaches to Stop Hypertension; aDASH. Alternate Dietary Approaches to Stop Hypertension. Two-way ANOVA analysis: S: differences according to sex; I: differences according to IR score; I: interaction between sex and IR. Differences between IR and non-IR within the same sex group (Student's t test or U de Mann-Whitney test depend the distribution of the variable): *p<0.05; ** p<0.01
