# Supplementary material for: Association of Healthy Eating Index-2015 and Dietary Approaches to Stop Hypertension Patterns with Insulin Resistance in Schoolchildren
Source: Nutrients. 2022 Oct 11;14(20):4232. doi: 10.3390/nu14204232 (PMC9607022; doi:10.3390/nu14204232)
Supplement: Supplementary file 1 [file nutrients-14-04232-s001.zip › nutrients-1929136-supplementary/nutrients-1929136-supplementary/Table S6. Diet quality in the schoolchildren studied according to HOMA-IR and age..pdf]

Table S6. Diet quality in the schoolchildren studied according to HOMA-IR and age

	8-10 years			11-13 years		
	**HOMA-IR≤3.16 (n=410)**	**HOMA-IR>3.16 (n=31)**	**p**	**HOMA-IR≤3.16 (n=399)**	**HOMA-IR>3.16 (n=14)**	**p**
Energy intake (kcal) #	2082±359	2161±479	0.205	**2191±386**	**2219±427**	**0.024**
Energy expenditure (kcal)	2105±347	2038±357	0.627	2120±356	1976±314	0.996
EI/EE (%)	103.8±23.3	98.7±28.6	0.329	**99.2±21.7**	**91.0±17.0** #	**0.030**
Proteins (%) -R	**15.4±2.3**	**16.5±1.7**	**0.017**	15.8±2.4	15.2±2.6	0.203
Carbohydrates (%)	41.1±5.1	40.1±5.3	0.429	40.9±5.0	40.5±4.9#	0.350
Lipids (%)	41.9±4.7	41.7±5.6#	0.433	41.7±4.8	42.5±4.9#	0.232
**HEI-2015 (total score)** #	59.6±8.3	59.2±8.6	0.427	58.7±8.9	56.3±8.6	0.097
Total Fruits (score)	3.7±1.4	3.5±1.7	0.701	3.6±1.5	3.7±1.5	0.830
Whole Fruits (score)	4.0±1.5	3.4±1.9	0.342	3.8±1.7	4.1±1.4	0.251
Total Vegetables (score)	3.1±1.4	3.0±1.4	0.660	3.1±1.3	2.8±1.6	0.421
Greens and Beans (score)	3.8±1.7	4.0±1.4	0.983	3.8±1.7	3.2±2.1	0.079
Whole Grains (score)	1.1±1.7	0.8±1.0	0.890	1.1±1.9	0.4±0.6	0.211
Dairy (score)	7.1±2.1	7.7±1.7	0.277	6.8±2.1	6.9±2.0	0.978
Total Protein Foods (score)-R	4.9±0.4	5.0±0.0	0.054	4.9±0.3	4.8±0.4	0.063
Seafood and Plant Proteins (score)	1.9±1.4	2.3±1.6	0.233	2.0±1.4	1.9±1.4	0.743
(PUFAs+MUFAs)/SFAs (score)	3.2±2.2	2.6±2.1	0.217	3.2±2.3	3.0±2.5	0.636
Refined Grains (score)	7.0±2.4	6.5±2.8	0.533	6.7±2.4	6.9±2.2	0.843
Sodium (score) -A	8.7±1.8	9.1±1.3	0.558	8.4±2.2	7.9±2.5	0.462
Added Sugars (score) -R	8.6±1.6	9.2±1.1	0.150	8.8±1.5	8.4±1.8	0.472
Saturated Fats (score)	2.3±2.1	2.1±1.8	0.664	2.5±2.2	2.2±1.9	0.721
Total Fruits (cups/1000kcal)	0.71±0.39	0.76±0.54	0.984	0.71±0.41	0.68±0.34	0.938
Whole Fruits (cups/1000kcal)	0.48±0.32	0.52±0.48	0.801	0.46±0.33	0.51±0.28	0.210
Total Vegetables (cups/1000kcal)	0.75±0.46	0.79±0.61	0.724	0.74±0.44	0.69±0.49	0.438
Greens and Beans (cups/1000kcal)	0.34±0.35	0.44±0.53	0.605	0.33±0.34	0.29±0.33	0.212
Whole Grains (oz /1000kcal)	0.17±0.27	0.12±0.15	0.889	0.17±0.29	0.06±0.10	0.211
Dairy (cups/1000kcal)	0.95±0.31	1.01±0.22 #	0.227	0.90±0.32	0.92±0.33	0.955
Total Protein Foods (oz /1000kcal) -R	**3.5±1.0**	**4.0±1.0** #	**0.028**	3.7±1.1	3.5±1.2	0.204
Seafood and Plant Proteins (cups /1000kcal)	0.69±0.54	0.91±0.74	0.228	0.74±0.61	0.68±0.51	0.744
(PUFAs+MUFAs)/SFAs	1.6±0.3	1.5±0.3	0.232	1.6±0.3	1.6±0.3 #	0.380
Refined Grains (oz/1000kcal)	2.5±0.7	2.6±0.7 #	0.214	2.6±0.7	2.6±0.6 #	0.427
Sodium (mg/1000kcal)	1152±222	1132±165	0.781	1198±263	1217±305	0.766
Added Sugars (% of kcal)	8.7±3.6	7.6±2.6	0.190	8.2±3.5	8.8±4.3	0.494
Saturated Fats (% of kcal)	14.4±2.1	14.7±1.9 #	0.289	14.3±2.2	14.8±2.5 #	0.157
**DASH (total score)**	23.5±3.7	23.8±3.1	0.760	22.9±3.8	23.5±4.0	0.510
Red meat (score)	3.0±1.4	3.0±1.5	0.934	2.8±1.4	3.3±1.5	0.108
Sugar drinks (score)	1.8±0.4	1.8±0.4	0.774	1.8±0.4	1.7±0.5	0.200
Sodium (score)	2.9±1.5	3.3±1.6	0.168	2.7±1.4	3.1±1.6	0.218
Whole Grains (score) -I	3.6±0.8	3.4±0.7	0.312	3.6±0.8	3.3±0.5	0.074
Low-fat dairy (score)	3.2±1.2	3.3±1.3	0.770	**3.2±1.2**	**3.8±1.0**	**0.013**
Vegetables (score)	3.0±1.4	2.7±1.4	0.449	3.0±1.4	2.9±1.7	0.651
Seeds. nuts and legumes (score)	3.0±1.4	3.2±1.3	0.545	2.9±1.5	3.0±1.6	0.777
Fruits (score)	3.0±1.4	2.9±1.4	0.843	3.1±1.5	2.6±1.3	0.141
Red meat (g)	101.2±46.5	106.3±54.5#	0.322	111.9±54.1	97.0±51.4#	0.089
Sugar drinks (g)	49.9±89.6	35.3±74.0	0.532	54.6±99.0	72.1±108.6	0.314
Sodium (mg)	2.416±574	2.306±523	0.423	2.531±669	2.385±651	0.251
Whole Grains (g)	10.1±16.0	6.8±8.2	0.836	10.2±17.9	3.6±5.8	0.186
Low-fat dairy (g)	97.1±130.1	119.8±152.8	0.977	**96.7±128.3**	**124.4±91.8**	**0.017**
Vegetables (g)	124.7±61.5	115.4±62.4	0.462	125.9±56.0	121.1±65.4	0.611
Seeds. nuts and legumes (g)	28.8±29.6	27.8±21.0	0.587	26.8±30.4	30.4±33.1	0.904
Fruits (g)	269.8±157.1	261.4±178.5	0.727	274.8±168.8	230.0±140.0	0.196

	8-10 years			11-13 years		
	**HOMA-IR≤3.16 (n=410)**	**HOMA-IR>3.16 (n=31)**	**p**	**HOMA-IR≤3.16 (n=399)**	**HOMA-IR>3.16 (n=14)**	**p**
**aDASH (total score)**	23.5±3.9	23.4±3.9	0.806	22.9±4.0	23.5±3.8	0.633
Red meat (score)	3.0±1.4	2.9±1.5	0.813	2.8±1.4	3.2±1.4	0.161
Sugar drinks (score) -A	1.8±0.4	1.9±0.3	0.382	1.8±0.4	1.7±0.5	0.118
Sodium (score)	2.9±1.5	3.0±1.4	0.791	2.7±1.5	2.7±1.7	0.994
Whole Grains (score)	3.6±0.8	3.4±0.7	0.365	3.6±0.8	3.3±0.5	0.132
Low-fat dairy (score)	3.2±1.2	3.3±1.3	0.713	**3.2±1.2**	**3.8±1.0**	**0.015**
Vegetables (score)	3.0±1.4	2.6±1.3	0.292	3.0±1.4	3.1±1.7	0.791
Seeds. nuts and legumes (score)	3.0±1.4	3.3±1.3	0.451	2.9±1.5	3.0±1.6	0.686
Fruits (score)	3.0±1.4	2.9±1.5	0.870	3.0±1.5	2.8±1.3	0.363
Red meat (g/1000kcal)	48.4±21.9	52.1±25.5	0.663	53.2±24.6	50.1±28.5	0.271
Sugar drinks (g/1000kcal)	23.7±44.0	15.6±32.7	0.512	25.9±47.6	35.2±51.5	0.325
Sodium (mg/1000kcal)	1152±222	1132±165	0.781	1198±263	1217±305	0.766
Whole Grains (g/1000kcal)	4.8±7.5	3.3±4.3	0.848	4.8±8.2	1.8±2.7	0.201
Low-fat dairy (g/1000kcal)	47.5±64.5	54.3±67.3	0.989	45.9±61.1	63.4±48.3	0.100
Vegetables (g/1000kcal)	59.4±27.9	54.3±23.6	0.500	60.2±27.1	63.7±37.8	0.926
Seeds. nuts and legumes (g/1000kcal)	13.7±14.1	13.5±9.0	0.445	12.6±14.1	15.6±16.9	0.722
Fruits (g/1000kcal)	128.8±73.1	127.8±88.7	0.779	130.8±79.6	116.5±64.7	0.473

HEI-2015. Healthy eating index; DASH. Dietary Approaches to Stop Hypertension; aDASH. Alternate Dietary Approaches to Stop Hypertension MUFAs, monounsaturated fatty acids; PUFAs, polyunsaturated fatty acids; SFAs, saturated fatty acids. Two-way ANOVA analysis: A: differences according to age; I: differences according to insulin resistance (IR) score; R: interaction between sex and IR. # Variable follow a normal distribution. For comparison of means, the Mann-Whitney U test was used if the distribution of the variables was not homogeneous, the Student's t-test for homogeneous distributions.
