# Supplementary material for: Association of Healthy Eating Index-2015 and Dietary Approaches to Stop Hypertension Patterns with Insulin Resistance in Schoolchildren
Source: Nutrients. 2022 Oct 11;14(20):4232. doi: 10.3390/nu14204232 (PMC9607022; doi:10.3390/nu14204232)
Supplement: Supplementary file 1 [file nutrients-14-04232-s001.zip › nutrients-1929136-supplementary/nutrients-1929136-supplementary/Table S7. Tertiles of the different indices according to sex.pdf]

Table S7. Tertiles of the different indices according to sex

	Girls	Boys
**HEI-2015**		
**T1**	36.3-56.7	31.3-54.7
**T2**	56.8-64.4	54.8-61.9
**T3**	64.5-85.9	61.9-82.2
**T2+T3**	56.8-85.9	54.8-82.2
**DASH**		
**T1**	12-22	14-21
**T2**	23-25	22-24
**T3**	26-35	25-33
**T2+T3**	23-35	22-33
**aDASH**		
**T1**	12-22	14-21
**T2**	23-25	22-24
**T3**	26-34	25-33
**T2+T3**	23-34	22-33
